# A retrospective study on the physical growth of twins in the first year after birth

**DOI:** 10.3389/fnut.2023.1168849

**Published:** 2023-09-22

**Authors:** Ting Pan, Yanru Huang, Qian Cheng, Li Chen, Yan Hu, Ying Dai, Xiao Liu, Zhiyang Jiang, Yuanfeng Zhong, Zhanzhan Zhang, Qian Chen, Qian Zhang, Xuan Zhang

**Affiliations:** ^1^Department of Child Health Care, Chongqing Growth, Development and Mental Health Center for Children and Adolescents, National Clinical Research Center for Child Health and Disorders, Ministry of Education Key Laboratory of Child Development and Disorders, Chongqing Key Laboratory of Child Health and Nutrition, Children’s Hospital of Chongqing Medical University, Chongqing, China; ^2^Department of Child Health Care, Luzhou People’s Hospital, Luzhou, Sichuan, China

**Keywords:** appropriate for gestational age, catch up growth, growth and development, physical anthropology, small for gestational age

## Abstract

**Objectives:**

This study analyzed the physical growth of small for gestational age (SGA) and appropriate for gestational age (AGA) twins up to one year after birth.

**Methods:**

Weight, length, and head circumference data of 0–1 year-old twins were collected from the Child Health Care System from 2010 to 2019. Physical data were presented as *Z*-scores. Five parameters – growth level of weight, body length, head circumference, growth velocity, and body proportion (weight for length) were compared in twins.

**Results:**

A total of 3,909 cases were collected (22.61% SGA, 77.39% AGA). 1. In both groups, WAZ (Weight for age *z*-score), HCZ (Head circumference for age *z*-score), and LAZ (Length for age *z*-score) increased more rapidly in the first 6 months. By one year of age, WAZ, HCZ, and LAZ had reached the normal range, but none had reached the average level of normal singleton children. 2. The mean values of WAZ, HCZ, and LAZ in the AGA group were between −1 and 0, and between −2 and − 1 in the SGA group, in the first year after birth. The SGA group lagged significantly behind the AGA group. The LAZ score of SGA and AGA was lower than the WAZ and HCZ scores. 3. The proportion of preterm AGA was the largest in twins, and the growth rate of preterm AGA was the fastest. Preterm twins had greater growth potential than term twins. However, the growth level of preterm SGA was always low. 4. The WFLZ (Weight for length *z*-score) in each group was approximately close to 0. The WFLZ of SGA was smaller than that of AGA twins at most time points. After 4 months of age, the WFLZ of twins had a downward trend. The WFLZ of preterm SGA approached −1 at approximately 1 year old.

**Conclusion:**

The physical growth of SGA and AGA in twins in the first year can reach the normal range but cannot reach the average level of normal singleton children. More attention should be paid to SGA in twins, especially preterm SGA. We should give proper nutritional guidance after 4 months of age to ensure the appropriate body proportion (weight for length) of SGA in twins.

**Clinical trial registration:**

www.chictr.org.cn, CTR2000034761.

## 1. Introduction

Small for gestational age (SGA) refers to children with birth weight or birth length below the 10th percentile for the same gestational age (GA) and sex. Some scholars have also defined SGA as less than P5 or P3 (1, 2). The International Society of Pediatric Endocrinology and the Growth Hormone Research Society jointly defined SGA as birth weight and/or birth length below mean-2SD for the same sex and the same gestational age (3). However, SGA in China is still determined as P10 with a birth weight below the same sex and the same gestational age (4). The incidence of SGA in China was 6.61% as of 2009 (5). The twin birth rate has increased in recent years, and the twin pregnancy rate in 2019 was 3.69%, according to the Special Committee on Twin Pregnancy of the Chinese Maternal and Child Health Association (6). There is a lack of relevant research on the physical growth and neuropsychological development of twins of appropriate for gestational age (AGA) and of small for gestational age (SGA), respectively.

Most SGA children will have catch-up growth, which can promote physical growth and neuropsychological development (7). The SGA children who fail to achieve catch-up growth are at risk for short stature, neuropsychological developmental problems, and behavioral problems in adulthood. However, excessive catch-up growth increases the risk of childhood overweight, obesity, hypertension (8), and metabolic syndrome in adulthood (9). Similar findings have been reported in twin studies (10, 11). In addition, catch-up growth is also observed in twins with AGA (12). How to balance the advantages and disadvantages of catch-up growth so that the SGA can reach the target height and weight without increasing the risk of metabolic syndrome in adulthood is a hot topic.

This study analyzed the physical growth of SGA and AGA in twins up to one year after birth, in order to provide a reference for promoting appropriate catch-up growth in twins.

## 2. Materials and methods

### 2.1. Data source

Study data were extracted from the Child Health Care System of the Children’s Hospital of Chongqing Medical University for 9,688 visits between January 2010 and November 2019. The data included physical measurements (head circumference, body length, and body weight) and demographic information (sex, gestational age, birth length, and weight). The inclusion criteria were: (1) children were twins. (2) age 0–1 month and follow-up to 11 months. The exclusion criteria were: missing key information and being unable to be contacted by phone.

### 2.2. Research groups

According to the 2015 birth weight standards for newborns with different GA in China (7), children with birth weights between P10 and P90 of the same sex and GA were placed in the AGA group, and those with birth weights below P10 were placed in the SGA group. Preterm infants were those with 28 weeks ≤ GA < 37 weeks and term infants were those with 37 weeks ≤ GA < 42 weeks. Participants were divided into 12 age groups based on their age in months. If two or more visits were made to the same child in the same age group, the average was taken.

### 2.3. Physical index evaluation standard

The *Z*-scores of the anthropometric data were calculated using WHO Anthro (version 3.2.2) software and included the following indicators as growth levels: weight for age *Z*-score (WAZ), head circumference for age *Z*-score (HCZ), and length for age *Z*-score (LAZ). In addition, the weight for length *Z*-score (WFLZ) was used to measure body proportions. Due to the small number of preterm infants in some age groups, we worked with chronological age in premature newborns and did not calculate the corrected *Z*-score.

### 2.4. Statistical analysis

The data were analyzed with SAS 9.4 software (SAS Institute) and tested for normality. The measurement data were statistically described by *X ± SD*. The difference between independent sample continuous variables was tested by an independent sample *T*-test. Multiple group comparisons were performed by Analysis of Variance (ANOVA). Counting data were analyzed using a Chi-squared test, and a non-parametric test was used for those variables that did not satisfy normal and equal variance. The growth trend of 0–to 11-month-old children was plotted using the hierarchical PROC SGPLOT (SAS 9.4) procedure. *p* < 0.05 indicated that the difference was statistically significant.

## 3. Results

A total of 3,909 cases of twins <1 year of age were enrolled. The data distribution is shown in Table 1. In the SGA group, 884 cases (22.61%) were enrolled, with 405 boys (10.36%) and 479 girls (12.25%). In the AGA group, there were 3,025 cases (77.39%), with 1,577 boys (40.34%) and 1,448 girls (37.04%). The proportion of preterm AGA (67.26%) was the highest. Birth weight in the SGA group was significantly lower than in the AGA group (2.00 ± 0.38 kg vs. 2.28 ± 0.48 kg, *p* < 0.001). The GA of the SGA group was significantly higher than that of the AGA group (36.05 ± 1.57 weeks vs. 34.54 ± 2.13 weeks, *p* < 0.001).

**Table 1:** General characteristics of SGA and AGA.

Variable		Full-term		Preterm	
		AGA	SGA	AGA	SGA
Cases, *n* (%)		396 (10.13)	378 (9.67)	2,629 (67.26)	506 (12.94)
Sex	Male infants, *n* (%)	230 (29.72)^a^	141 (18.22)	1,347 (42.97)^b^	264 (8.42)
	Female infants, *n* (%)	166 (21.45)	237 (30.62)	1,282 (40.89)	242 (7.72)
Birth weight (kg)		2.85 ± 0.26^c^	2.28 ± 0.23	2.20 ± 0.44^c^	1.79 ± 0.33
Gestational age (weeks)		37.21 ± 0.52^c^	37.44 ± 0.73	34.15 ± 1.97^c^	35.01 ± 1.17

^a^Sex ratio of full-term AGA compared with full-term SGA, *p* < 0.001; ^b^Sex ratio of preterm AGA compared with preterm SGA, *p* = 0.7402; ^c^Compared with SGA, *p* < 0.05.

### 3.1. Growth level and growth rate of SGA and AGA twins

**Figure 1:**
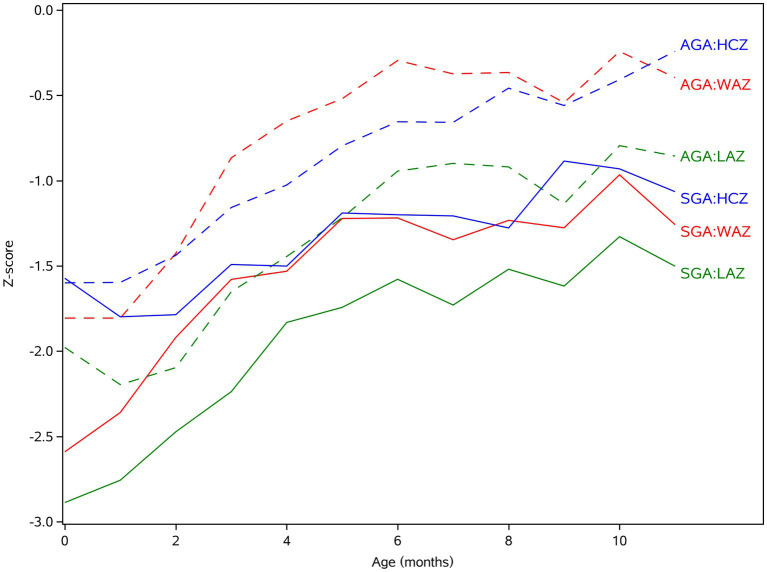
The *Z*-score of anthropometric parameters of SGA and AGA in twins under 1 year old. WAZ, weight for age *Z*-score; LAZ, length for age *Z*-score; HCZ, head circumference for age *Z*-score.

Table 2; Figure 1 show the mean and trend of WAZ, LAZ, HCZ of SGA, and AGA in twins from 0 to 11 months, which indicate: 1. The WAZ, LAZ, and HCZ of twins gradually increased within 1 year. The WAZ and LAZ of AGA twins within 6 months old and those indicators of SGA twins within 5 months old had a relatively rapid growth velocity, then those indicators of both two groups slowed down after 5-6 months. 2. Except for the age of 0 months, the WAZ, LAZ, and HCZ of SGA in twins were lower than those of AGA in twins at most time points (*p* < 0.05). LAZ was lower than WAZ and HCZ within 1 year after birth in all groups. 3. At the age of 11 months, the growth levels of AGA and SGA in twins were still lower than those of mean value of singleton children (*Z*<0) but still in the normal range (*Z* > −2); the WAZ, HCZ, and LAZ of the AGA group were > −1, and the WAZ, HCZ, and LAZ of the SGA group ranged from −2 to approximately −1.

**Table 2:** WAZ, LAZ, and HCZ of SGA and AGA in twins under 1 year of age (X ± S).

Age (m)	AGA				SGA			
	*n*	WAZ	LAZ	HCZ	*n*	WAZ	LAZ	HCZ
0	24	−1.94 ± 1.18*	−2.14 ± 1.40*	−1.73 ± 1.23*	4	−2.59 ± 1.33	−2.89 ± 0.89	−1.57 ± 0.67
1	285	−1.86 ± 1.20	−2.26 ± 1.30	−1.64 ± 1.17*	81	−2.39 ± 1.16	−2.79 ± 1.22	−1.82 ± 1.02
2	371	−1.44 ± 1.35	−2.11 ± 1.36	−1.45 ± 1.27	87	−1.92 ± 1.28	−2.47 ± 1.29	−1.79 ± 1.13
3	334	−0.87 ± 1.26	−1.65 ± 1.28	−1.16 ± 1.23	111	−1.58 ± 1.23	−2.24 ± 1.21	−1.49 ± 1.14
4	360	−0.65 ± 1.19	−1.44 ± 1.26	−1.03 ± 1.21	95	−1.53 ± 1.31	−1.83 ± 1.29	−1.50 ± 1.21
5	323	−0.52 ± 1.18	−1.22 ± 1.12	−0.80 ± 1.16	105	−1.22 ± 1.34	−1.74 ± 1.08	−1.19 ± 1.11
6	346	−0.30 ± 1.14	−0.94 ± 1.09	−0.65 ± 1.10	105	−1.22 ± 1.18	−1.58 ± 1.05	−1.20 ± 1.17
7	201	−0.37 ± 1.11	−0.90 ± 1.05	−0.66 ± 1.13	74	−1.35 ± 1.15	−1.73 ± 1.14	−1.21 ± 1.12
8	266	−0.37 ± 1.15	−0.92 ± 1.08	−0.46 ± 1.15	70	−1.23 ± 1.33	−1.52 ± 1.21	−1.28 ± 1.13
9	181	−0.54 ± 1.16	−1.14 ± 1.19	−0.56 ± 1.28*	51	−1.28 ± 1.01	−1.62 ± 0.92	−0.89 ± 0.96
10	185	−0.24 ± 1.14	−0.79 ± 1.08	−0.41 ± 1.06	50	−0.97 ± 1.04	−1.33 ± 0.95	−0.93 ± 1.18
11	157	−0.40 ± 1.12	−0.86 ± 1.07	−0.24 ± 1.12	52	−1.26 ± 1.28	−1.50 ± 1.27	−1.06 ± 0.95

WAZ, weight for age *Z*-score; LAZ, length for age *Z*-score; HCZ, head circumference for age *Z*-score; *Compared with SGA group *p* ≥ 0.05, others *p* < 0.05.

### 3.2. Growth level of preterm and full-term SGA and AGA twins

Table 3 shows the changes in physical indicators in the full-term and preterm AGA groups. The number of preterm AGA infants was larger than that of full-term AGA twins. WAZ, LAZ, and HCZ gradually increased after birth in AGA twins. At most time points, the WAZ, HCZ, and LAZ of preterm AGA twins were smaller than those of full-term AGA subjects. There was no significant difference in the WAZ and HCZ at 7 months and in the WAZ, HCZ, and LAZ at 8–10 months between preterm AGA and full-term AGA twins. All the indicators were between −1 and 0 in both groups at 11 months.

**Table 3:** WAZ, LAZ, HCZ of full-term and preterm AGA in twins under 1 year of age (X ± S).

Age (m)	Full-term AGA				Preterm AGA			
	*n*	WAZ	LAZ	HCZ	*n*	WAZ	LAZ	HCZ
0	3	−0.67 ± 0.14*	−1.27 ± 0.44	−0.64 ± 0.82	22	−2.12 ± 1.15	−2.26 ± 1.45	−1.88 ± 1.21
1	43	−0.85 ± 0.89*	−1.29 ± 0.72*	−0.77 ± 1.05*	247	−2.03 ± 1.17	−2.42 ± 1.30	−1.79 ± 1.12
2	34	−0.64 ± 1.10*	−1.13 ± 0.69*	−0.58 ± 0.94*	338	−1.52 ± 1.35	−2.21 ± 1.37	−1.54 ± 1.26
3	50	−0.06 ± 0.82*	−0.74 ± 0.83*	−0.37 ± 0.89*	284	−1.02 ± 1.27	−1.82 ± 1.28	−1.30 ± 1.24
4	41	−0.24 ± 1.05*	−0.75 ± 0.82*	−0.36 ± 0.92*	319	−0.70 ± 1.20	−1.53 ± 1.28	−1.11 ± 1.22
5	49	0.00 ± 1.10*	−0.51 ± 0.92*	−0.20 ± 0.80*	274	−0.61 ± 1.17	−1.34 ± 1.11	−0.90 ± 1.18
6	48	0.16 ± 0.90*	−0.37 ± 1.05*	−0.21 ± 0.91*	298	−0.37 ± 1.16	−1.04 ± 1.07	−0.73 ± 1.12
7	27	−0.12 ± 1.07	−0.26 ± 0.92*	−0.30 ± 0.94	174	−0.41 ± 1.11	−1.00 ± 1.04	−0.71 ± 1.15
8	36	−0.28 ± 1.18	−0.79 ± 0.83	−0.20 ± 1.17	230	−0.37 ± 1.14	−0.93 ± 1.12	−0.50 ± 1.14
9	24	−0.85 ± 1.39	−1.10 ± 1.45	−0.86 ± 1.24	157	−0.50 ± 1.12	−1.14 ± 1.15	−0.51 ± 1.28
10	23	−0.03 ± 0.98	−0.51 ± 0.86	−0.03 ± 1.14	162	−0.27 ± 1.15	−0.83 ± 1.10	−0.46 ± 1.04
11	19	0.02 ± 1.10*	−0.15 ± 1.00*	−0.23 ± 1.17	138	−0.45 ± 1.12	−0.95 ± 1.05	−0.24 ± 1.12

*Compared to preterm AGA, *p* < 0.05.

Table 4 shows the changes in physical indicators in the full-term and preterm SGA groups. In both groups, there was an increasing trend in WAZ, LAZ, and HCZ. The indicators in the preterm SGA group were significantly smaller than those of the full-term SGA group at most time points, except that there was no significant difference in LAZ between the two groups at 9–11 months. All the indicators in preterm SGA were at a low level (−2 <*Z*< −1) at 11 months old.

**Table 4:** WAZ, LAZ, and HCZ of full-term and preterm SGA in twins under 1 year of age (X ± S).

Age (m)	Full-term SGA				Preterm SGA			
	*n*	WAZ	LAZ	HCZ	*n*	WAZ	LAZ	HCZ
0	1	−0.61 ± 0.00	−1.73 ± 0.00	−0.64 ± 0.00	3	−3.25 ± 0.22	−3.27 ± 0.54	−1.88 ± 0.29
1	34	−1.81 ± 1.05*	−2.23 ± 1.15*	−1.23 ± 1.00*	48	−2.80 ± 1.05	−3.20 ± 1.12	−2.23 ± 0.82
2	34	1.42 ± 0.95	−1.82 ± 0.93	−1.18 ± 0.74	53	−2.23 ± 1.37	−2.87 ± 1.33	−2.16 ± 1.17
3	50	−1.00 ± 0.92*	−1.67 ± 0.83*	−0.94 ± 0.78*	61	−2.05 ± 1.25	−2.70 ± 1.28	−1.94 ± 1.20
4	42	−1.12 ± 1.18*	−1.26 ± 0.90*	−1.13 ± 1.17*	53	−1.86 ± 1.32	−2.28 ± 1.38	−1.80 ± 1.16
5	52	0.65 ± 1.25	−1.27 ± 0.92	−0.7 ± 0.78	53	−1.78 ± 1.18	−2.2 ± 1.02	−1.67 ± 1.18
6	48	−0.81 ± 1.10*	−1.17 ± 0.84*	−0.85 ± 0.92*	57	−1.56 ± 1.14	−1.92 ± 1.09	−1.50 ± 1.27
7	31	−0.83 ± 1.11*	−1.08 ± 1.00*	−0.75 ± 0.88*	43	−1.72 ± 1.03	−2.20 ± 1.02	−1.54 ± 1.17
8	28	−0.87 ± 1.34	−1.27 ± 1.16	−1.00 ± 1.37	42	−1.48 ± 1.28	−1.68 ± 1.24	−1.46 ± 0.91
9	15	−0.71 ± 1.04*	−1.28 ± 0.94	−0.49 ± 0.78	36	−1.51 ± 0.91	−1.76 ± 0.89	−1.05 ± 0.99
10	22	−0.58 ± 1.13*	−1.06 ± 1.02	−0.38 ± 1.09*	28	−1.27 ± 0.85	−1.54 ± 0.86	−1.36 ± 1.08
11	22	−0.81 ± 0.99*	−1.16 ± 0.96	−0.71 ± 0.78*	30	−1.59 ± 1.39	−1.75 ± 1.42	−1.32 ± 1.00

*Compared to preterm SGA, *p* < 0.05.

**Figure 2:**
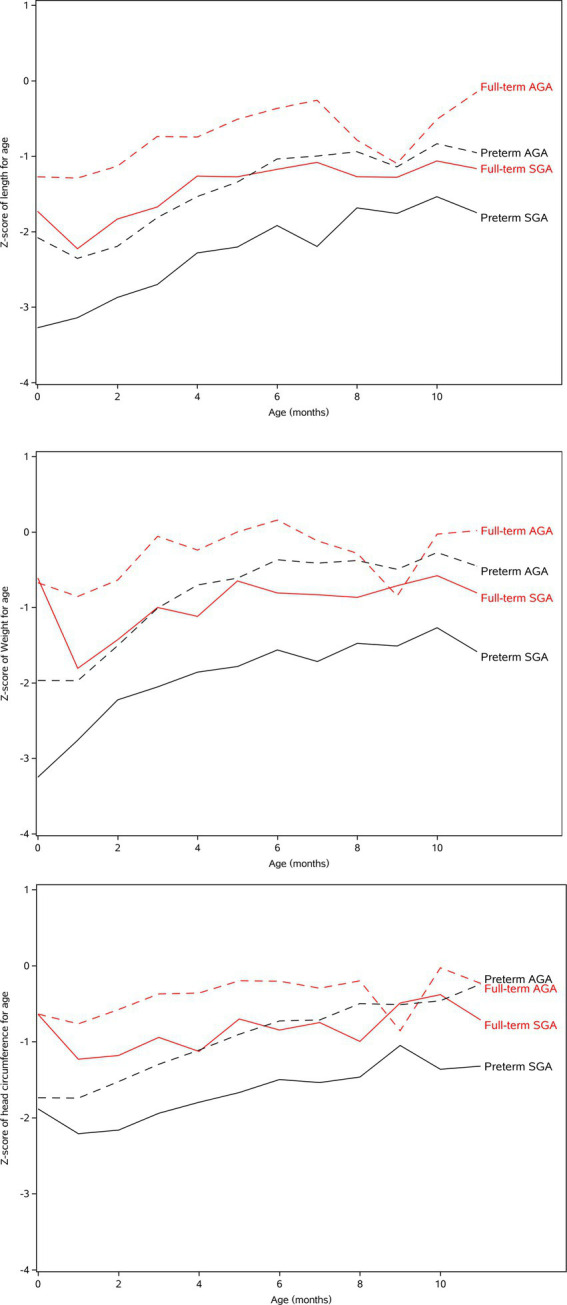
The WAZ, LAZ, and HCZ of preterm AGA, full-term AGA, preterm SGA, and full-term SGA in Twins.

Figure 2 shows the trends of WAZ, LAZ, and HCZ from 0 to 11 months in four groups. It can be seen that the trend of WAZ, LAZ, and HCZ is consistent. Preterm SGA and preterm AGA in twins grew rapidly after birth; the growth potential of preterm twins was greater than that of full-term twins. The growth velocity was faster before the age of 5–6 months, then slower. In preterm infants, there was a significant lag behind full-term infants in physical growth during the first year. Preterm SGA continuously had a lower growth level. The changes in WAZ, LAZ, and HCZ of full-term AGA and full-term SGA were slower in the first year after birth.

### 3.3. Body proportions of SGA and AGA twins

**Figure 3:**
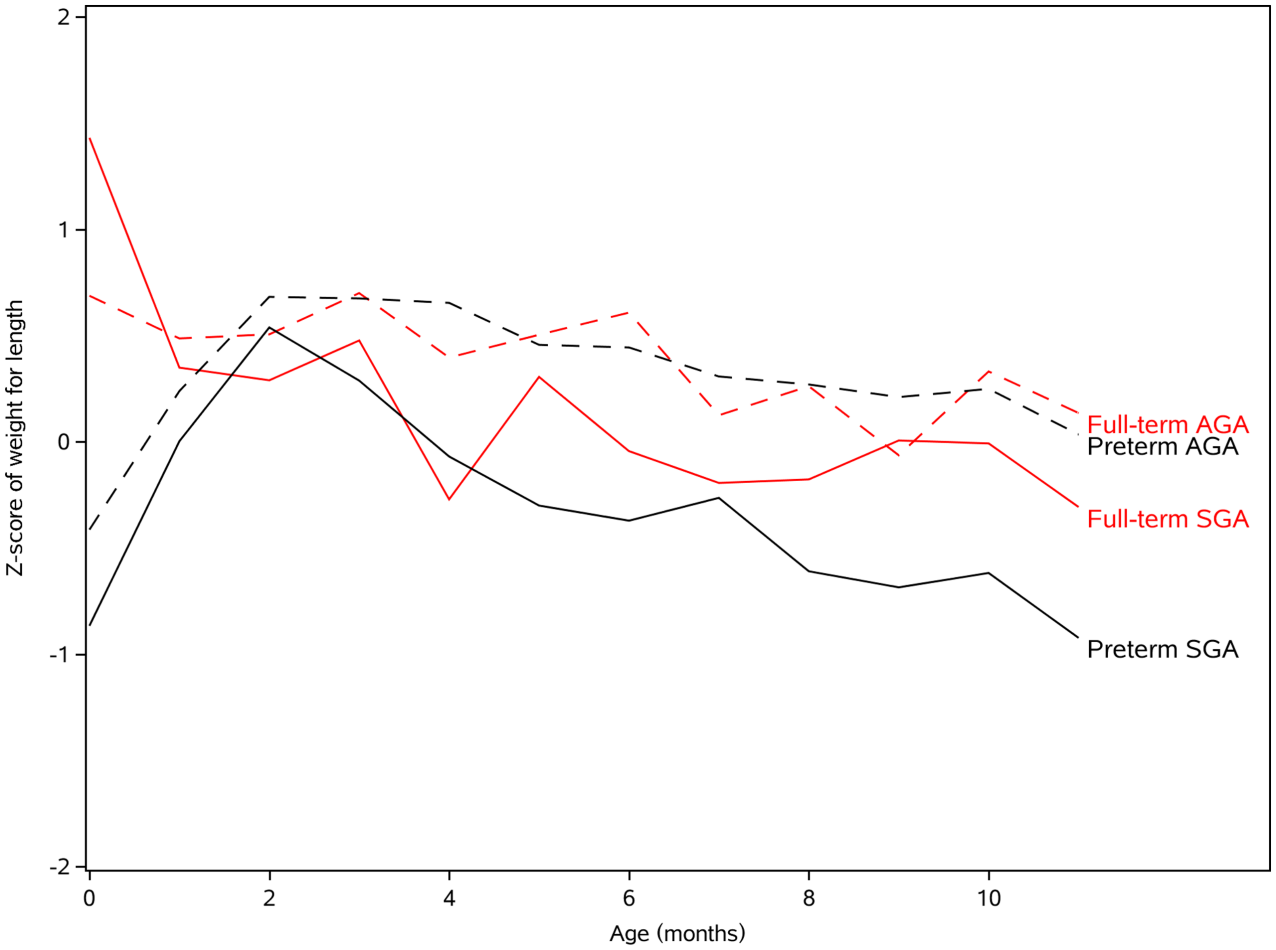
The *Z*-score of weight for length (WFLZ) of preterm AGA, full-term AGA, preterm SGA, and full-term SGA in twins.

As shown in Table 5; Figure 3, the WFLZ were similar and the *Z* scores were close to 0 in the first year; the WFLZ of SGA was smaller than that of AGA at most time points, showing a statistically significant difference at 3 months of age. The WFLZ of full-term SGA and preterm SGA did not increase significantly but decreased after 1 month of age, and WFLZ<0 after 4 months of age in full-term and preterm SGA, with the WFLZ of preterm SGA gradually approaching -1 It was suggested that after SGA experiences early accelerated catch-up growth, attention should be paid to nutritional intake after 4 months of age to ensure appropriate body proportions, especially in preterm SGA.

**Table 5:** WFLZ of preterm AGA, full-term AGA, preterm SGA, and full-term SGA in twins (^−^X ± S).

Age (m)	Full-term AGA	Preterm AGA	Full-term SGA	Preterm SGA	*p*
0	0.69 ± 0.63	0.06 ± 2.50	1.43 ± 0.00	−0.87 ± 0.44	0.1416
1	0.49 ± 0.96	0.43 ± 1.67	0.35 ± 0.97	0.21 ± 1.74	0.1165
2	0.51 ± 1.19	0.71 ± 1.16	0.27 ± 0.99	0.54 ± 1.29	0.1861
3	0.70 ± 0.90	0.67 ± 0.93	0.48 ± 0.96	0.29 ± 0.93	0.0192
4	0.40 ± 1.04	0.65 ± 0.96	−0.27 ± 1.22	−0.07 ± 0.82	<0.001
5	0.50 ± 1.00	0.46 ± 1.05	0.30 ± 1.2	−0.3 ± 1.06	<0.001
6	0.61 ± 0.72	0.44 ± 1.07	−0.04 ± 1.06	−0.37 ± 1.10	<0.001
7	0.12 ± 0.96	0.31 ± 1.07	−0.19 ± 0.94	−0.26 ± 0.99	<0.05
8	0.26 ± 1.14	0.27 ± 1.02	−0.18 ± 1.19	−0.61 ± 1.20	<0.001
9	−0.06 ± 0.97	0.21 ± 0.96	0.01 ± 1.00	−0.69 ± 0.96	<0.001
10	0.33 ± 0.94	0.25 ± 1.04	−0.01 ± 1.01	−0.62 ± 0.80	<0.001
11	0.13 ± 0.97	0.04 ± 1.03	−0.31 ± 1.04	−0.92 ± 1.11	<0.001

*p* is the comparison among the four groups by Analysis of Variance (ANOVA).

## 4. Discussion

The main conclusions of this study are as follows: 1. At approximately 1 year of age, the growth level of twins could reach the normal range but did not reach the average level of singleton children. The growth level of SGA in twins lagged behind that of AGA within 1 year of birth. The LAZ of SGA and AGA was lower than that of WAZ and HCZ. 2. The proportion of preterm AGA was the largest in twins, and the growth rate was the fastest. Preterm twins had greater growth potential than term twins. However, the growth level of preterm SGA was always low. 3. The WFLZ in each group was approximately close to 0. The WFLZ of SGA is smaller than that of AGA at most time points. After reaching 4 months of age, the WFLZ of twins had a downward trend. The WFLZ of preterm SGA approached −1 at about 1 year of age.

### 4.1. Comparing SGA and AGA growth velocity

#### 4.1.1. Comparison of AGA and SGA

In the present study, we found that the physical indices of twins increased significantly but were still lower than the average level of singleton children at 11 months. This may be related to the low birth weight of twins. A study (13) on the physical growth of twins aged 0–4 years with birth weight discordance in twins showed that the growth level of low birth weight twins lagged behind that of normal birth weight twins. A study (11) of monozygotic twins suggested that birth weight affects not only recent physical growth and development, but also further secondary development at puberty, and hence final height. Another study (14) has shown that catch-up growth after birth is not associated with birth weight but with intrauterine growth restriction (IUGR), especially when IUGR happens at 20-32 weeks of gestational age. A study (15) on singletons showed that the mean body weight and length of AGA with IUGR were lower than those of regular AGA and that the gap was not significant at 4 months of age. This suggests that fetuses with IUGR may develop better catch-up growth after birth, especially in AGA. which may be related to higher insulin sensitivity, lower leptin levels, and lower body fat percentage (16).

This study found that the growth level of SGA twins was always lower than that of AGA twins, which is the same as that found in another study on singletons (14). In addition, this study also found that all groups had a smaller LAZ than WAZ and HCZ, suggesting that it takes a longer time to achieve length catch-up. A nationwide Japanese (17) population-based study showed that 15% of 32,533 full-term SGA twins did not show catch-up growth in height until 2 years of age, which is the same conclusion as previous singleton studies (14, 18). A Dutch study (19) showed a faster growth rate in twins after birth, although they did not reach the same height and weight until 2.5 years of age. A study (18) of 9 cities in China found that the growth patterns of infants aged 1 to 12 months under different feeding patterns were very similar, which revealed that the growth differences of infants under different feeding patterns were much smaller than the growth differences of infants under different ethnic and region. That is, recent nutrition has less impact on length catch-up than long-term nutrition and genetic factors.

#### 4.1.2. Comparison of full-term and preterm twins

In this study, the proportion of premature AGA twins was the highest and their growth velocity was the fastest. It is suggested that better catch-up growth occurs in premature AGA. The results are consistent with Anchieta et al. (20), who concluded that catch-up growth in preterm infants after delivery is closely related to birth weight. EJ McLaughlin et al. (15) showed that, compared with AGA with regular intrauterine growth rate, AGA with growth restriction had lower *Z*-scores in body weight, body length, and BMI; the difference was not significant at 4 months of age. This suggests that preterm AGA subjects may achieve catch-up growth earlier and faster than SGA infants.

On the other hand, preterm SGA has always had a lower growth level. In a singleton children study, Nagasaka et al. (21) found that preterm infants were twice as likely as full-term infants to have short stature at 3 years of age, while preterm SGA infants were 4.5 times more likely to be of short stature than full-term infants. This may be related to the risk of gastrointestinal disorders such as necrotizing enterocolitis and food allergy in premature infants. In addition, the rapid transition to breast milk after birth may result in an insufficient supply of protein and energy; the introduction time and quantity of supplementary foods and the high incidence of feeding intolerance may also lead to decreased growth in premature infants. Full-term twins will more easily achieve catch-up growth than normal singleton children due to more mature organs, phylogeny, and better nutrient uptake.

### 4.2. Body proportion is an important indicator of having appropriate catch-up growth or not

Another issue that needs to be addressed is the prevention of excessive catch-up growth to reduce the risk of childhood excess weight, obesity, hypertension, and the risk of developing type 2 diabetes, cardiovascular disease, and metabolic syndrome in adulthood. Therefore, we need to pay attention to the change in body proportion (expressed as WFLZ in this study). In this study, the WFLZ values in the early postnatal period were similar and in the normal range (close to 0) in all groups. In singleton studies, BMI increased faster at 0 to 3 months after birth for preterm SGA than for full-term SGA and AGA twins; it increased with age but decreased after 8 months of age (22, 23). In the present study, the WFLZ of SGA decreased gradually after 4 months of age. It is suggested that nutritional intake after 4 months of age should receive more attention so as to ensure body shape proportion after early accelerated catch-up growth of SGA, especially for premature SGA infants.

The 2019 Infant Feeding and Nutrition Guidelines (24) state that breastfeeding should be the first choice for SGA feeding and that the choice of feeding method should be based on gestational age rather than birth weight. For full-term SGA, regular use of the preterm formula is not recommended to promote better growth. However, this study suggests that for twins with SGA, close monitoring of growth and individualized nutritional guidance are necessary if intensive feeding is required. For twins with AGA, attention should be paid to monitoring physical growth to avoid adverse long-term outcomes caused by overfeeding.

## 5. Conclusion

In summary, the physical growth of twins during the first year of life may be more complex than that of singletons. Twins can achieve normal range, which is above mean-2SD in the first year after birth but may not reach the average level of singleton children of the same age. The growth level of twins with SGA lags behind that of AGA, especially preterm SGA, so more attention should be paid to SGA in twins. After achieving catch-up growth in the early stage of SGA, we should give proper nutritional guidance after 4 months of age to ensure the appropriate body proportion of SGA twins. Although the number of participants in the study was relatively large, there were only a few participants in some age groups, particularly in the case of SGA and full-term AGA children. More children should be enrolled and followed up regularly in future studies.

## Data availability statement

The datasets generated and analyzed during the current study are not publicly available due to patients’ privacy concerns but are available from the corresponding author upon reasonable request.

## Ethics statement

The studies involving humans were approved by the Institutional Review Board of Children’s Hospital of Chongqing Medical University (CHCMU). The studies were conducted in accordance with the local legislation and institutional requirements. The ethics committee/institutional review board waived the requirement of written informed consent for participation from the participants or the participants’ legal guardians/next of kin because The present study was an observational and retrospective study, it did not need to use human biological specimens. We applied for the exemption from the need for informed consent and was reviewed and approved by the Institutional Review Board of Children’s Hospital of Chongqing Medical University (CHCMU). The Institutional Review Board of CHCMU waived the need for informed consent.

## Author contributions

XZ conceptualized and designed the study, acquired funding, conducted the data analysis, and reviewed and revised the manuscript. TP and YrH drafted the initial manuscript and revised it. QCg, LC, YH, YD, XL, ZJ, YZ, ZZ, QC, and QZ acquired the data and were responsible for the follow-up of these children. All authors approved the final version of this manuscript and agreed to its publication.
